# Advances in sulodexide-based long-term anticoagulation for a myasthenia gravis patient with giant thymoma

**DOI:** 10.3389/fphar.2025.1543612

**Published:** 2025-02-26

**Authors:** Zhou Liu, Liang Zhang, Wei Peng, Qianqian Chen, Yanguang Hou, Liying Zhan, Guang Li

**Affiliations:** ^1^ Department of Intensive Care Unit, Renmin Hospital of Wuhan University, Wuhan, China; ^2^ Department of Radiology, Renmin Hospital of Wuhan University, Wuhan, China

**Keywords:** sulodexide, myasthenia gravis, venous thrombosis, anticoagulation, anti-thrombosis

## Abstract

This case report describes a geriatric male patient with myasthenia gravis (MG) secondary to giant thymoma, presenting with progressive muscle weakness and ptosis. The diagnosis of MG was confirmed through pathology, imaging, and laboratory evaluations. Considering the significant surgical risks associated with the giant thymoma, adjuvant chemotherapy was initiated. Unfortunately, 2 weeks following chemotherapy, the patient developed acute respiratory failure and sudden loss of consciousness. Emergency endotracheal intubation was performed, and he was then transferred to the intensive care unit (ICU) and treated with immunoglobulin, plasmapheresis, prednisone, and pyridostigmine. During ICU hospitalization, the patient developed severe lower limb edema accompanied by increased skin temperature, particularly on the left side. Ultrasound imaging confirmed extensive thrombosis in the left iliac and femoral veins, with thrombosis involving 50%–67% of the venous lumen. To prevent the risk of pulmonary embolism (PE), an inferior vena cava filter was implanted, and low-molecular weight heparin (LMWH) was prescribed for anticoagulation. Unfortunately, the patient later experienced intermittent melena and heparin-induced thrombocytopenia (HIT), with hemoglobin levels decreasing to 55 g/L and platelet counts decreasing to 57 × 10^9^/L. Given the adverse events associated with LMWH, sulodexide (SDX) was substituted as a novel anticoagulant with multiple benefits, including reduced thrombosis and bleeding risk, anti-inflammatory effects, and vascular endothelium protection. SDX demonstrated excellent efficacy and safety, with no adverse effects observed during the 3-year follow-up period. In conclusion, SDX should be considered an ideal potential option for long-term anticoagulation in patients with complex conditions such as MG with both thrombotic and bleeding risks.

## Introduction

Myasthenia gravis (MG) is an autoimmune disease primarily mediated by acetylcholine receptor (AchR) antibodies, with contributions from cell-mediated immune response and complement activation. Although AchR antibodies are the most common pathogenic antibodies, other postsynaptic membrane antibodies, including muscle-specific receptor tyrosine kinase (MuSk), low-density lipoprotein receptor-related protein 4 (LRP-4), and ryanodine receptor (RyR), also play significant roles in pathogenesis (Wiendl et al., 2023). Currently, the primary therapies for MG include cholinesterase inhibitors, immune suppressants, and targeted biological therapies such as belimumab, zilucoplan, and efgartigimod, along with plasma exchange and surgery (Gilhus and Verschuuren, 2015).

Patients with MG are predisposed to hypercoagulability and thrombotic complications (Müller and Schwenke, 1971), highlighting the critical need to identify effective and safe long-term anticoagulation strategies. MG patients are particularly vulnerable to developing venous thrombotic diseases, including cerebral infarction, myocardial infarction, lower limb venous thrombosis, and even pulmonary embolism (PE) (Lin et al., 2018; Steg and Lefkowitz, 1994; Mizrahi et al., 2009). Currently, the most commonly used anticoagulants are unfractionated heparin (UFH), low-molecular weight heparin (LMWH), vitamin K antagonists, and novel oral anticoagulants such as rivaroxaban and dabigatran (Vitiello and Ferrara, 2023). However, heparin is frequently associated with complications, including heparin-induced thrombocytopenia (HIT), gastrointestinal bleeding, skin or visceral bleeding, and, in high-risk patients, even intracranial hemorrhage (Warkentin, 2015). These complications underscore the need for alternative anticoagulation therapies that offer high efficacy with minimal adverse effects.

In the case presented, an elderly male MG patient, who developed lower limb thrombosis and was being treated with LMWH, suffered from HIT and gastrointestinal hemorrhage under continuous monitoring. Based on existing literature reports and accumulated clinical experience, sulodexide (SDX) was substituted as the long-term anticoagulation strategy (Pompilio et al., 2021). Remarkably, during the entire 3-year follow-up, no thrombotic or hemorrhagic adverse events were observed during SDX therapy. Characterized by diverse biochemical activities, SDX proved to be an ideal choice for patients with complex embolism. To date, there is limited literature on the application of SDX for long-term anticoagulation in MG patients, particularly for those with remarkable thrombotic complications. This case report provides the first documented evidence of SDX’s safety and efficacy in managing MG-associated venous thrombotic complications over the long-term observation period.

## Case report

**FIGURE 1:**
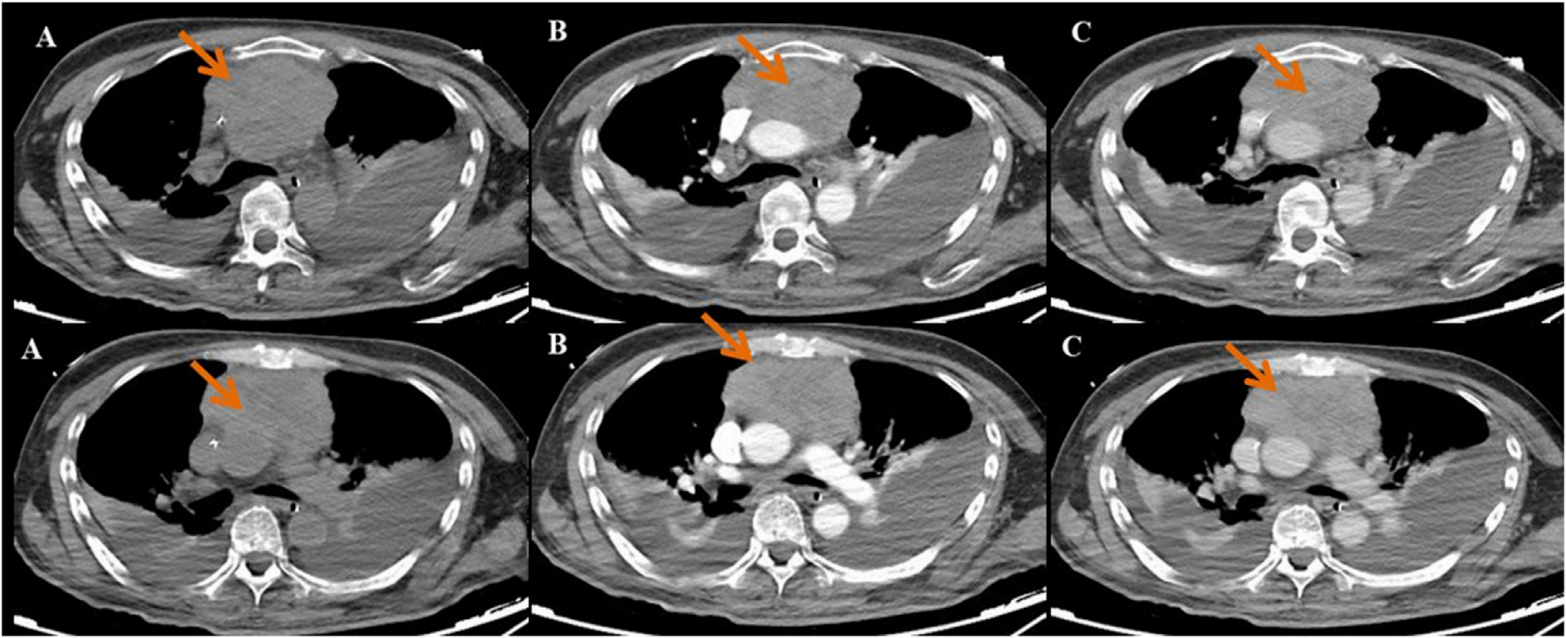
The enhanced chest CT indicated that the size of the huge thymoma is 8.7 × 9.0 cm (red arrow). **(A)** Plain scan sequence; **(B)** arterial phase; and **(C)** venous phase.

**FIGURE 2:**
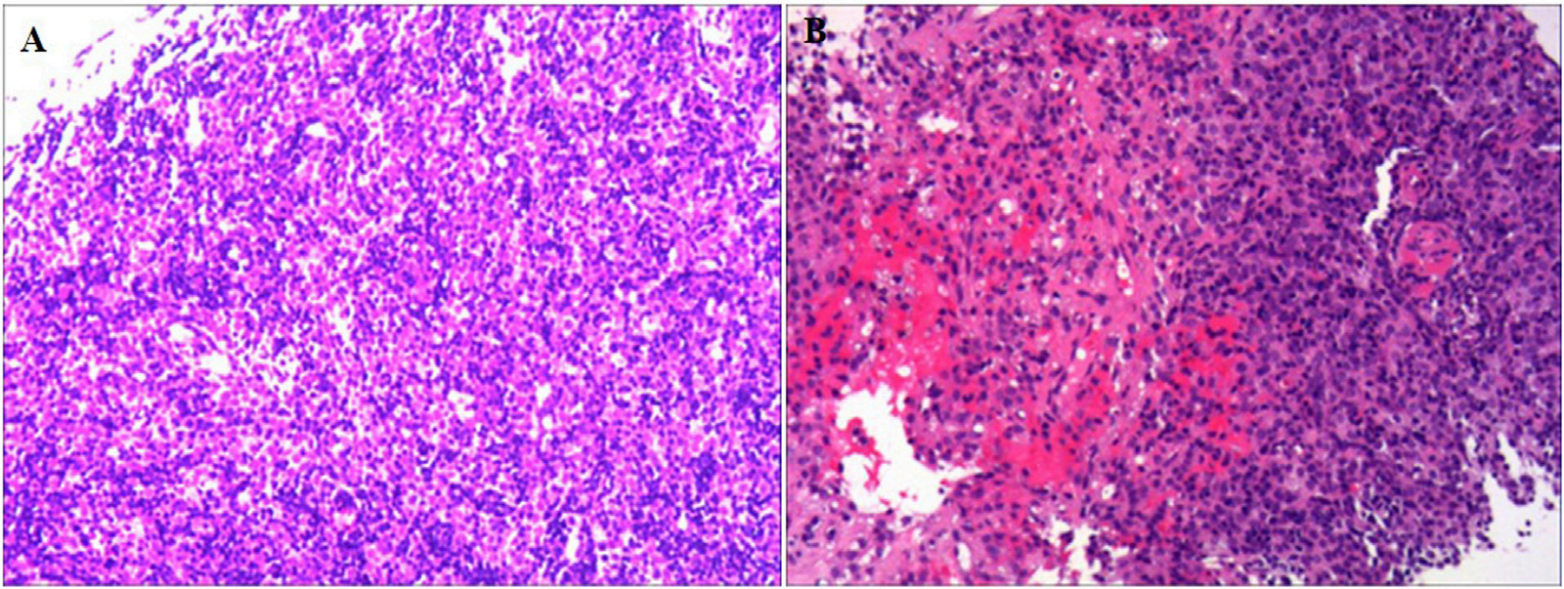
Pathological staining of thymoma tissue. **(A)** HE staining (×100 times) and **(B)** HE staining (×30 times).

A 60-year-old male was admitted to the neurology department on 1 October 2020, with complaints of eyelid drooping and muscle weakness. Initially, the patient experienced difficulty in opening eyes and fatigue, which progressively worsened into fluctuating muscle weakness. More recently, he deteriorated to respiratory distress and occasional dysphagia. The patient had no significant medical history in the past. Physical examination revealed swallowing dysfunction (Wada drinking test level IV), ptosis, and restricted eye movements. Laboratory examination on October 1st showed that the blood routine, cardiac function, renal function, liver function, and coagulation profiles were all within normal limits. Serological antibodies, conducted on October 2nd, revealed elevated AChR antibody levels (11.589 nmol/L), but the results for both MuSK and LRP-4 antibodies were negative. An enhanced chest CT examination on 3 October revealed a large thymoma in the anterior mediastinum, measuring 8.7 cm × 9.0 cm (Figure 1). Pathology examination of thymoma on 9 October confirmed malignant thymoma (type B2) with CD3 (+), CD5 (+), and Ki67 (+, 80%) staining (Figure 2). A PET-CT examination on 13 November showed abnormal and heterogeneous radioactive distribution with an irregular and large soft tissue mass, considered thymoma, measuring approximately 9.0 cm × 6.3 cm × 8.7 cm. The clinical characteristics of the case report are summarized in Table 1.

**TABLE 1:** Clinical summary of a 60-year-old male patient with malignant thymoma and myasthenia gravis.

Category	Details
Patient information	Age: 60; sex: male; admission date: 1 October 2020
Presenting symptoms	Eyelid drooping, muscle weakness, difficulty opening eyes, fatigue, fluctuating muscle weakness, respiratory distress, and occasional dysphagia
Past medical history	No significant medical history
Physical examination	Swallowing dysfunction (WADA drinking test level IV), ptosis, and restricted eye movements
Serological antibody (2 October)	Elevated AChR antibody levels (11.589 nmol/L) and negative MuSK and LRP-4 antibodies
Laboratory findings (1 October)	Normal blood routine, cardiac function, renal and liver function, and coagulation profiles
Radiology (3 October)	Enhanced chest CT: large thymoma (8.7 cm × 9.0 cm) in the anterior mediastinum
Pathology (9 October)	Needle biopsy of thymoma: malignant thymoma (type B2), CD3 (+), CD5 (+), and Ki67 (+, 80%)
PET-CT (13 November)	Heterogeneous radioactive distribution, irregular soft tissue mass with uneven density, and thymoma 9.0 cm × 6.3 cm × 8.7 cm

The patient’s symptoms, auto-antibody profile, radiological images, and pathological results were completely consistent with the 2022 diagnostic criteria for MG (González-Larrocha et al., 2017). Despite treatment with pyridostigmine and prednisone, there was no significant improvement in the patient’s condition. Given the surgical risks associated with giant malignant thymoma, the multidisciplinary team recommended neoadjuvant chemotherapy combined with immunotherapy, including paclitaxel (200 mg on d1 and d8), carboplatin (500 mg on d1, q3w), and pembrolizumab (200 mg on d1, q3w). Following two weeks of chemotherapy, the patient suddenly progressed to severe circulatory and respiratory failure, necessitating emergency tracheal intubation and transfer to the intensive care unit (ICU). The patient deteriorated to a myasthenic crisis, and high doses of immunoglobulin, corticosteroids, plasmapheresis (five sessions), and mechanical ventilation (MV) were administered. Gradually, the patient condition improved. Simultaneously, early rehabilitation and physical therapy were also initiated for recovery.

**FIGURE 3:**
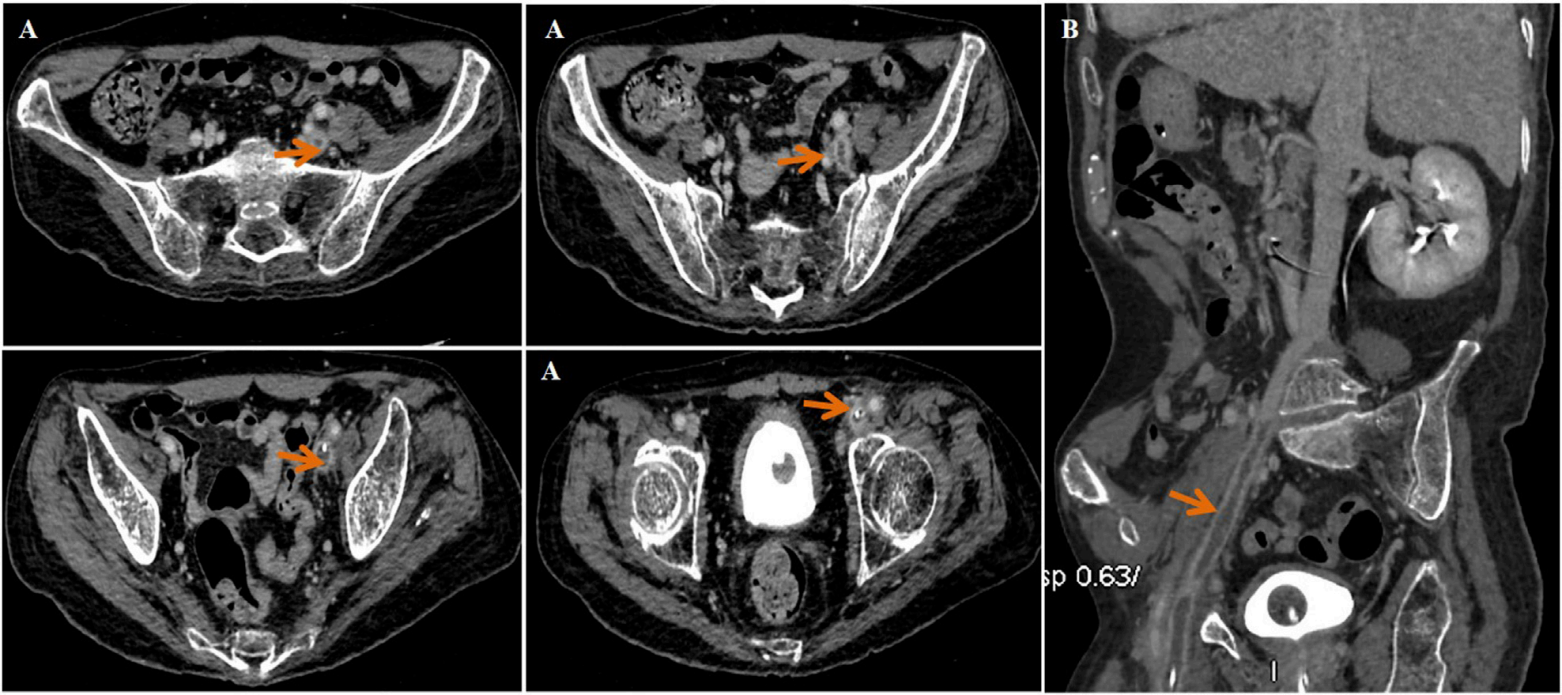
Magnetic resonance angiography (MRA) indicated thrombosis in the left femoral vein, internal and external iliac veins, and common iliac vein (red arrow). **(A)** Transverse plane and **(B)** sagittal plane.

**FIGURE 4:**
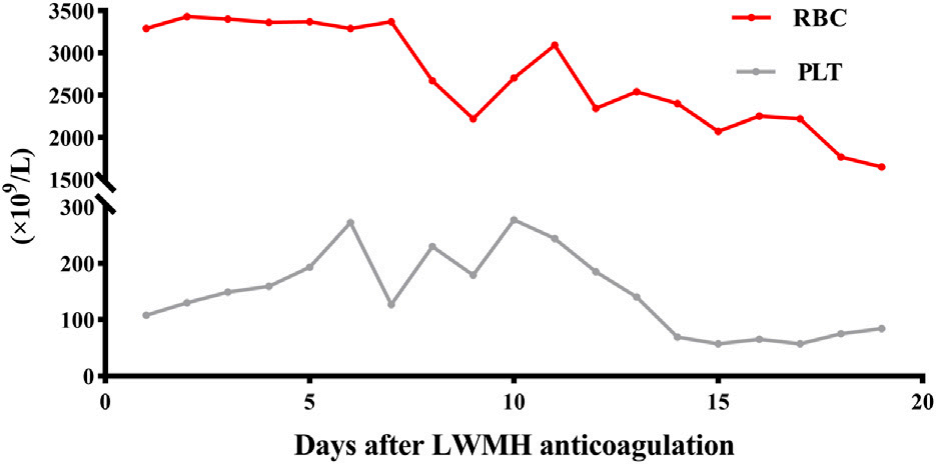
RBC and PLT of the MG patient during LMWH anticoagulation. RBC, red blood cell (10^12^per/L); PLT, platelet (10^9^per/L).

Approximately 3 months later, the patient developed moderate swelling in lower limbs, accompanied by a slightly increased skin temperature, suggesting the possibility of venous thrombosis. Ultrasound confirmed severe thrombosis in the left iliac and femoral veins, with approximately 50%–67% occlusion of the lumen. Thus, low-molecular weight heparin (LMWH) (4100Axa qd) was initially prescribed as the standard anticoagulation regimen but failed to halt the progression of thrombosis. Follow-up magnetic resonance angiography (MRA) revealed substantial filling defects, exceeding two-thirds of the lumen, in the left femoral vein, internal iliac veins, external iliac veins, and common iliac vein (Figure 3). To prevent the risk of PE, an inferior vena cava filter was promptly implanted. Postoperatively, LMWH (4100Axa q12h) was resumed regularly to reduce the risk of in-stent restenosis. Approximately 2 months later, the patient presented with massive melena and HIT, with the hemoglobin level rapidly decreasing to as low as 55 g/L and platelet count sharply decreasing to 57 × 10^9^/L (Figure 4). The anticoagulant-related coagulation dysfunction further exacerbated gastrointestinal hemorrhage risk, prompting the immediate suspension of the LMWH regimen (Supplementary Table S1).

**FIGURE 5:**
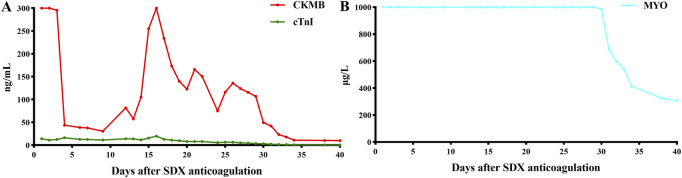
Dynamic changes in myocardial enzymes and liver function when SDX is taken as anticoagulation therapy. **(A)** CKMB and ctnI dynamic changes and **(B)** MYO dynamic changes. CKMB, creatine kinase MB (ng/mL); MYO, myoglobin (μg/L); cTnI, cardiac troponin I (ng/mL).

**FIGURE 6:**
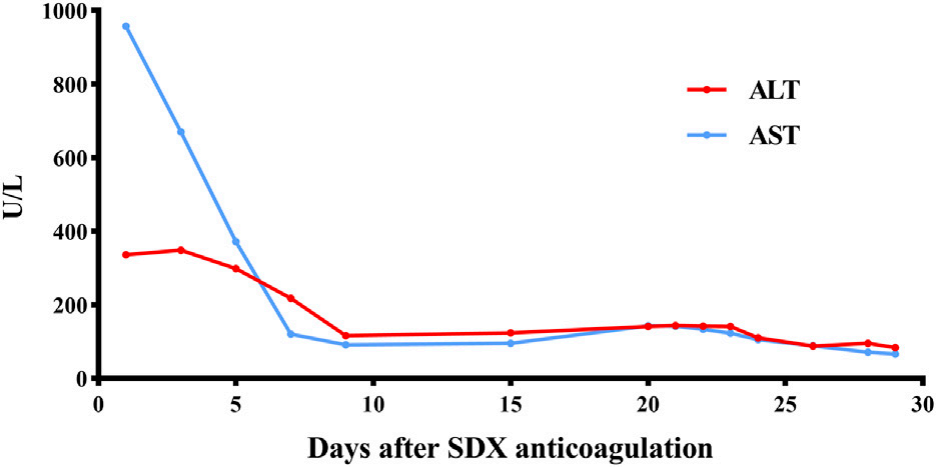
Dynamic changes in ALT and AST when SDX is taken as anticoagulation therapy. ALT, glutamic pyruvic transaminase (U/L); AST, glutamic oxaloacetic transaminase (U/L).

Unexpectedly, vein thrombosis recurred in the lower limbs of the hypercoagulability MG patient during the suspension of anticoagulation after several months. Under this circumstance, SDX was introduced as a substitute anticoagulant for this complex embolism and hypercoagulable MG patient. The diverse biochemical properties of SDX contributed to achieve thrombus stability without causing further bleeding or embolism complications. Regular and dynamic monitoring of blood routine, liver and renal function, cardiac function, and coagulation parameters showed gradual improvement throughout the whole SDX anticoagulation period (Figures 5, 6).

**FIGURE 7:**
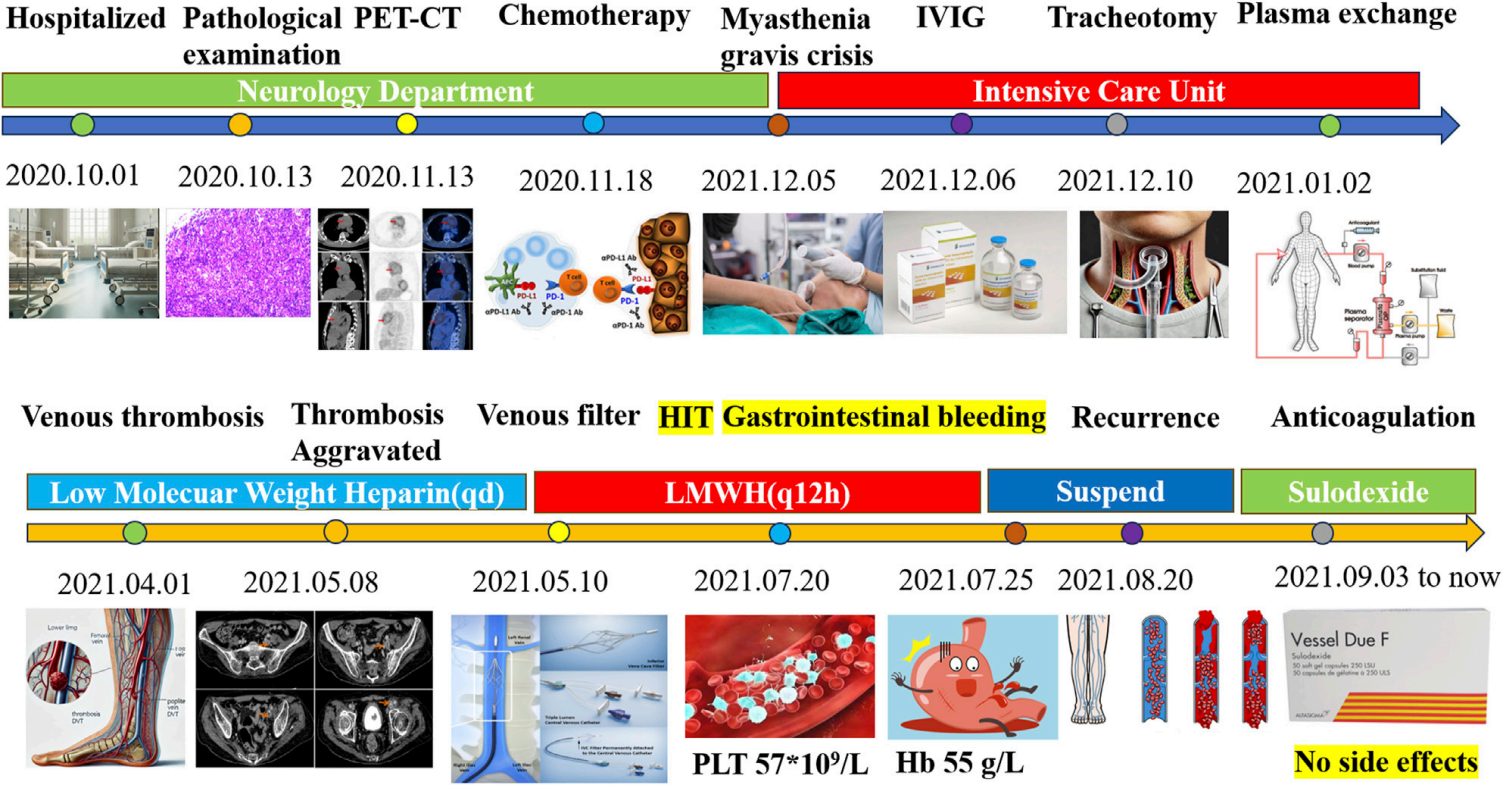
Summary of therapy of the myasthenia gravis patient and anticoagulation management. PET-CT, positron emission tomography/computed tomography; IVIG, intravenous immunoglobulin; HIT, heparin-induced thrombocytopenia; LMWH, low-molecular weight heparin; PLT, platelet.

Currently, the patient remains bedridden in the neurology department for years, requiring tracheotomy-assisted oxygen inhalation and intermittent sputum aspiration. Throughout the follow-up period, biannual radiology, quarterly ultrasounds, and fortnightly coagulation function confirmed the absence of new thrombotic or hemorrhagic events. Notably, the patient never experienced myocardial infarction, deep vein thrombosis (DVT), cerebral infarction, cerebral hemorrhage, or gastrointestinal bleeding during the prolonged period of SDX anticoagulation (Figure 7).

## Discussion

### Hypercoagulability in MG

In China, the prevalence of MG is approximately 4.09 per 1,000,000, slightly higher than in Europe and the United States (Bubuioc et al., 2021). Hypercoagulability is a prominent feature of MG, and the necessity of an anticoagulation strategy should not be ignored. Several factors, including MG-related immune dysregulation, infections, endothelial damage, medications, and thymic abnormalities, are closely linked to the hypercoagulability state. *a. Endothelial damage*: neuromuscular-specific antibodies as part of the autoimmune process may indirectly damage the endothelium, promoting the hypercoagulable state. *b. Inflammation*: recurrence infections and/or myasthenic crises can trigger a massive release of inflammatory mediators such as interleukin and tumor necrosis factor (TNF), which can damage the vascular endothelium and contribute to the hypercoagulable condition (Epaulard et al., 2015; Weng et al., 2024). *c. Drug effects*: common MG medications, including pyridostigmine (Leong et al., 2009), cyclophosphamide (Krüger-Genge et al., 2023), glucocorticoids (Johannesdottir et al., 2013), analgesics, and sedatives (Haroutiunian et al., 2009), as well as immunoglobulin (Jin et al., 2020), may alter coagulation dynamics. Cyclophosphamide may cause myelosuppression and thrombocytopenia, accelerating the thrombosis process (Krüger-Genge et al., 2023). Glucocorticoids significantly increase the risk of venous thromboembolism (Johannesdottir et al., 2013). Analgesics and sedative drugs such as opioids and benzodiazepines may interact with other drugs used to treat MG, affecting the coagulation function (Haroutiunian et al., 2009). *d. Thymic abnormalities*: MG is usually accompanied by thymic disease, with 80% of patients presenting with thymic abnormalities, including thymoma in 10%–20% of cases (Bernard et al., 2016). These thymic abnormalities influenced coagulation through immune modulation (Müller and Schwenke, 1971). *e. Thymoma secretion*: malignant thymomas can secrete platelet-activating factors and other procoagulant substrates, thereby accelerating the thrombosis process (Bui et al., 2020).

To investigate the hypercoagulability in MG and thymoma patients, we reviewed the relevant published case reports. Seven cases were identified who were diagnosed with either MG or thymoma in combination with thrombotic disease (Lin et al., 2018; Bui et al., 2020; Cohen et al., 2024; San Norberto et al., 2018; Lin et al., 2020; Ball and Cocks, 2015; Berbecar et al., 2017). Among these, three patients had upper deep vein thrombosis (42.86%), one patient had lower venous thrombosis (14.29%), one patient had PE (14.29%), one patient had rare renal vein thrombosis (14.29%), and one patient (14.29%) was unclear about the location of DVT. The anticoagulation drugs included Eliquis, rivaroxaban, warfarin, LMWH, and enoxaparin. Notably, 14.29% of the patients experienced a recurrence of thrombotic disease (Table 2).

**TABLE 2:** Case report of myasthenia gravis/thymoma causing thrombosis disease.

Author	Age	Gender	Country	Primary disease	Thrombosis	Treatment	Outcome
Cohen NE	69 years	Female	Bradenton, United States	Myasthenia gravis	Upper extremity deep vein thrombosis	Anticoagulation (Eliquis)	Remission
San Norberto EM	60 years	Male	Valladolid, Spain	Myasthenia gravis	Deep vein thrombosis	Anticoagulation (rivaroxaban)	Remission
Lin S	45 years	Female	Xining, China	Myasthenia gravis	Pulmonary embolism	Anticoagulation (warfarin)	Remission
Lin CY	71 years	Male	Tainan, Taiwan	Myasthenia gravis	DVT in the left femoral and popliteal veins	Anticoagulation (LMWH)	Remission
Ball SL	Unknown	Unknown	Sunderland, United Kingdom	Thymoma	DVT in the left upper limb	Anticoagulation (rivaroxaban)	Recurrence
Bui H	38 years	Male	Texas, United States	Thymoma	Bilateral upper extremity deep vein thrombosis	Anticoagulation (enoxaparin)	Remission
Berbecar VT	68 years	Male	Bucharest, Romania	Thymoma	Inferior vena cava and renal vein thrombosis	Anticoagulation (oral anticoagulants)	Remission

### Comparison of sulodexide and LMWH

Both SDX and LMWH are polysaccharides derived from glycosaminoglycans (GAGs) extracted from intestinal mucosa, and both have long-standing clinical applications (Tran et al., 2022). However, SDX distinguishes itself from LMWH in several key aspects. To begin with, SDX combines both anticoagulant and anti-inflammatory properties with a minimal risk of HIT, making it a suitable choice for a long-term strategy. In contrast, LMWH is more prone to HIT, especially with prolonged management. Second, SDX consists of 80% fast-moving heparin (FMH) and 20% dermatan sulfate (DS), providing a synergistic effect. However, LMWH relies solely on FMH for anticoagulant properties (Masola et al., 2014). Third, SDX is absorbed and distributed more efficiently than LMWH. It has a peak plasma time of just 15 min and exhibits 14 times greater volume distribution than LMWH (Luzzi et al., 2014). Additionally, whether administered intravenously, intramuscularly, or orally, SDX can be absorbed and widely distributed, exhibiting high bioavailability. Fourth, SDX demonstrates superior endothelial affinity, inhibiting platelet aggregation, reducing fibrinogen levels, and promoting systemic fibrinolytic and thrombolytic activity. Unlike LMWH, the longer sugar chain of SDX allows it to inhibit both anti-thrombin-III (AT-III) and factor-IIa (FIIa), directly reducing thrombin production and platelet activation. *In vitro* experiments show that SDX reduces platelet aggregation by one-fifth compared to enoxaparin, demonstrating significant antithrombotic activity (Siddiqui et al., 2020). Recent studies confirmed that SDX significantly reduces the recurrence of venous thromboembolism while maintaining a low risk of bleeding (Walenga et al., 2016; Ligi et al., 2020). Furthermore, SDX not only reduced the risk of post-thrombotic syndrome (PTS) but also promoted ulcer healing, highlighting the advantages of managing thrombotic diseases (Pompilio et al., 2022). In contrast, LMWH primarily inhibits factor Xa (FXa), which blocks the pathway of thrombin production. However, it has a limited effect on already formed thrombin.

**FIGURE 8:**
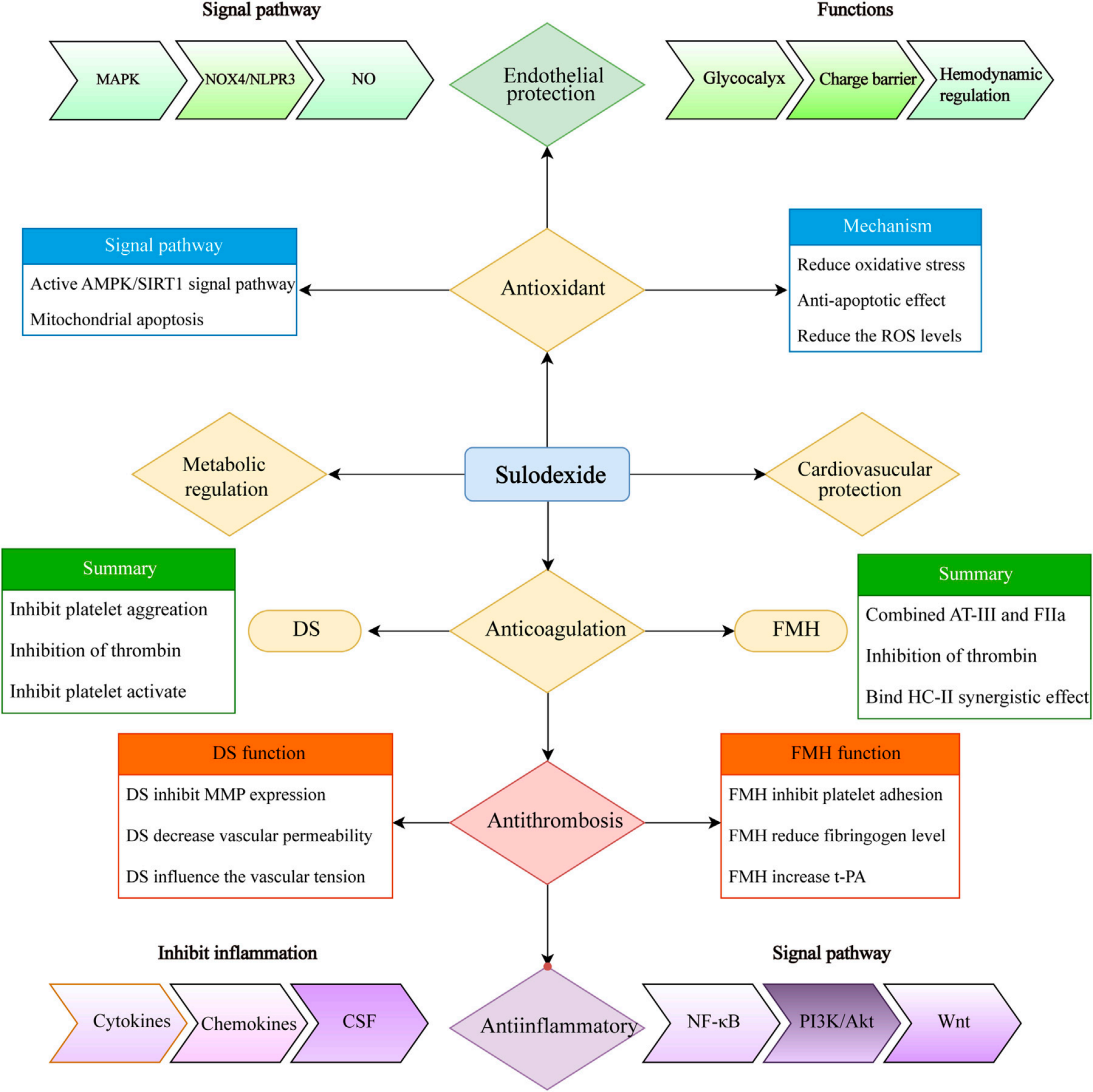
Summary of sulodexide’s multiple biochemical activities (including anti-coagulation, anti-thrombosis, anti-inflammatory, antioxidant, endothelial and cardiovascular protection, and metabolic regulation). MAPK, mitogen-activated protein kinase; NOX4, NADPH oxidase 4; AMPK, AMP-activated protein kinase; SIRTs, sirtuins; ROS, reactive oxygen species; FMH, fast-moving heparin; DS, dermatan sulfate; MMP, matrix metalloproteinase; t-PA, tissue plasminogen activator; CSF, colony-stimulating factor; PI3K, phosphatidylinositol 3-kinase; Akt, protein kinase B.

SDX has demonstrated a broad range of biological activities, such as modulating inflammation and endothelial protection. It can inhibit the secretion of inflammatory cytokines (e.g., IL-1β, IL-7, IL-8, IL-12, and IL-17), chemokines, and colony-stimulating factors (CSFs) through the NF/κB signal pathway, thereby reducing oxidative stress and promoting the restoration of endothelial glycocalyx integrity (Raffetto et al., 2020). It can also alleviate oxidative stress and endothelial apoptosis caused by oxygen and glucose deprivation (Gabryel et al., 2016). By interacting with the PI3K/Akt signaling pathway, SDX can effectively eliminate excessive oxidative stress and toxic substances. The endothelial protection is largely attributed to interactions with glycocalyx, a crucial component of the endothelial surface. SDX not only serves as the raw material for glycocalyx synthesis but also aids in the restoration of impaired endothelial cells and the extracellular matrix, thereby maintaining the balance of the microvascular function (Ying et al., 2023). Additionally, the DS components of SDX suppress matrix metalloproteinase (MMP-9) activity, preserving endothelial permeability and the extracellular matrix (Ying et al., 2023). SDX has also been shown to inhibit platelet activation and aggregation, slowing down the thrombus formation and significantly reducing thrombus volume (Barbanti et al., 1992). Proteomic analysis revealed that key activators in the KEGG pathway, including von Willebrand factor (vWF), coagulation factor (FV), and vitamin k-dependent protein C (PC) were upregulated in response to SDX (Li et al., 2023). SDX, with its overall antithrombotic effect, also contributes to the activation of the fibrinolytic system and inhibition of platelet aggregation. Beyond its antithrombotic properties, SDX offers additional benefits, such as reducing the risk of PTS (38), promoting ulcer healing (Carroll et al., 2019), and improving outcomes in peripheral arterial disease (PAD) (Sosińska-Zawierucha et al., 2018). Furthermore, SDX has favorable metabolic effects, including the reduction in triglycerides and cholesterol levels (Li et al., 2015; Crepaldi et al., 1990). A summary of SDX bioactivity and mechanism is presented in Figure 8.

### Clinical implications and future directions

For MG patients with thrombotic complications and bleeding risks, individualized anticoagulation strategies are crucial. SDX presents a promising alternative, owing to its unique combination of anti-coagulant, anti-inflammatory, endothelial protective, and metabolic regulatory properties. SDX has demonstrated significant efficacy in cardiovascular protection, endothelial restoration, anti-inflammation effects, anti-oxidative activity, and inhibition of platelet aggregation. It has already been widely used to manage a variety of arteriovenous thrombosis (Carroll et al., 2019; Gaddi et al., 2020), PTS (Murai, 2024), cardiovascular and cerebrovascular diseases (Condorelli et al., 1994), chronic kidney disease (Stefoni et al., 2014), diabetes (Bignamini et al., 2021), and even COVID-19-associated coagulopathy (Szolnoky and González-Ochoa, 2022). In the future, SDX may emerge as a valuable therapeutic option for expanding indications for more patients.

### Conclusion

These findings highlight the importance of personalized anticoagulation strategies in complex patients, particularly those with MG, who are prone to thrombotic events but also face heightened bleeding risk. SDX, with its dual anticoagulant and anti-inflammatory properties, emerges as a promising alternative to conventional therapies. In this case, the absence of adverse events during SDX therapy suggests its potential as a safety choice for long-term anticoagulation in high-risk patients. Future large-scale studies are warranted to further confirm its efficacy and assess its broader applications in thrombotic complications in MG and other complex conditions.

In summary, SDX exerts a broad range of biological effects, including antithrombotic, anti-thrombosis, pro-fibrinolytic, anti-inflammatory, and endothelial protective effects, as well as metabolic regulation and cardiovascular protection. It has emerged as a potential therapeutic option for managing chronic complex venous insufficiency, including venous ulceration, and preventing recurrent venous thromboembolism, with a low risk of massive bleeding or recurrence. Given its favorable profile, SDX may represent a promising alternative for long-term anticoagulation in MG patients. A Bayesian network meta-analysis has also confirmed that SDX is effective in reducing bleeding and mortality from various causes, such as thromboembolism (VTE), PE, myocardial infarction, and stroke, positioning it as a promising alternative for extended anticoagulation strategies (Pompilio et al., 2020). However, large-scale trials and extended follow-up are necessary to further confirm its efficacy and broader applications, such as transplantation surgery, in-stent restenosis, membranous nephropathy, and ischemia-reperfusion injury.

### Limitations

Although SDX offers clear advantages, its effects may vary across different populations due to genetic, racial, and environmental factors. This single-case report highlights its potential in the MG patient, thus limiting the ability to generalize the findings to a larger population. Therefore, further studies, including randomized controlled trials, are essential to confirm SDX efficacy and safety in diverse populations.
