# Supplementary material for: Advances in sulodexide-based long-term anticoagulation for a myasthenia gravis patient with giant thymoma
Source: Front Pharmacol. 2025 Feb 26;16:1543612. doi: 10.3389/fphar.2025.1543612 (PMC11900545; doi:10.3389/fphar.2025.1543612)
Supplement: Supplementary file 1 [file Table1.docx]

**Supplement Table 1** Changes in blood routine and coagulation function in MG patient during LMWH anticoagulation

| **Variable** | **WBC**  (10^9^per/L) | **Neu**  (%) | **RBC**  (10^12^per/L) | **Hb**  (g//L) | **PLT**  (10^9^per/L) | **PT**  (sec) | **APTT**  (sec) | **INR** | **D-dimer**  (mg/L) | **AT-III**  (%) |
| --- | --- | --- | --- | --- | --- | --- | --- | --- | --- | --- |
| Day01 | 4.57 | 47.70 | 3.29 | 109 | 108 | 13.7 | 31.3 | 1.19 | 0.45 | 94.4 |
| Day02 | 4.51 | 43.30 | 3.43 | 112 | 130 | 13.5 | 30.0 | 1.17 | 0.33 | 112.2 |
| Day03 | 5.55 | 53.50 | 3.40 | 112 | 149 | 13.7 | 31.9 | 1.19 | 0.76 | 79.5 |
| Day04 | 5.61 | 51.10 | 3.36 | 110 | 159 | 12.8 | 37.4 | 1.11 | 1.80 | 78.8 |
| Day05 | 12.22 | 73.70 | 3.37 | 111 | 193 | 13.0 | 32.7 | 1.12 | 2.97 | 101.5 |
| Day06 | 9.41 | 80.60 | 3.29 | 110 | 272 | 11.3 | 33.5 | 1.47 | 3.97 | 87.0 |
| Day07 | 10.51 | 73.00 | 3.37 | 102 | 127 | 16.3 | 32.0 | 1.42 | 2.06 | 83.1 |
| Day08 | 9.94 | 77.40 | 2.67 | 98 | 230 | 14.1 | 39.7 | 1.22 | 1.23 | 83.4 |
| Day09 | 6.43 | 65.10 | 2.22 | 84 | 179 | 12.3 | 33.4 | 1.06 | 3.84 | 89.4 |
| Day10 | 9.07 | 86.04 | 2.70 | 90 | 277 | 12.3 | 31.8 | 1.06 | 1.01 | 90.3 |
| Day11 | 9.37 | 82.10 | 3.09 | 102 | 244 | 16.2 | 33.2 | 1.41 | 1.06 | 125.8 |
| Day12 | 8.39 | 65.90 | 2.34 | 76 | 185 | 11.9 | 29.8 | 1.33 | 1.08 | 101.7 |
| Day13 | 5.85 | 62.10 | 2.54 | 84 | 140 | 12.8 | 30.7 | 1.51 | 1.16 | 78.4 |
| Day14 | 4.68 | 62.20 | 2.40 | 79 | 69 | 16.6 | 31.3 | 1.68 | 1.13 | 87.4 |
| Day15 | 5.32 | 73.30 | 2.07 | 67 | 57 | 18.2 | 27.8 | 1.44 | 0.45 | 94.4 |
| Day16 | 7.74 | 81.40 | 2.25 | 74 | 65 | 17.1 | 33.5 | 1.39 | 0.33 | 112.2 |
| Day17 | 4.53 | 69.30 | 2.22 | 72 | 57 | 19.7 | 32.3 | 1.29 | 0.56 | 89.5 |
| Day18 | 3.38 | 62.70 | 1.77 | 59 | 75 | 18.5 | 30.0 | 1.37 | 0.67 | 98.3 |
| Day19 | 3.55 | 51.80 | 1.65 | 55 | 84 | 18.7 | 31.9 | 1.49 | 0.78 | 87.9 |

**WBC**: White blood cell (10^9^per/L); **RBC**: Red blood cell (10^12^ per/L); **Neu**: Neutrophils (%); **Hb**: Hemoglobin (g/L); **PLT**: Platelet (10^9^per/L); **PT**: Prothrombin time (sec); **APTT**: activated partial thromboplastin time (sec); **INR**: international normalized ratio; **AT-Ⅲ:** antithrombin Ⅲ (%)
